# Advances in Nanotechnology as a Potential Alternative for Plant Viral Disease Management

**DOI:** 10.3389/fmicb.2022.935193

**Published:** 2022-06-30

**Authors:** Pranab Dutta, Arti Kumari, Madhusmita Mahanta, K. K. Biswas, Agnieszka Dudkiewicz, D. Thakuria, Abdelrazek S. Abdelrhim, S. Basanta Singh, Gomathy Muthukrishnan, K. G. Sabarinathan, Mihir K. Mandal, N. Mazumdar

**Affiliations:** ^1^School of Crop Protection, College of Post Graduate Studies in Agricultural Sciences, Central Agricultural University (Imphal), Imphal, India; ^2^Division of Plant Pathology, Indian Agricultural Reserach Institute (IARI), New Delhi, India; ^3^Department of Dietetics and Food Analysis, Faculty of Science and Technology, Jan Dlugosz University in Czestochowa, Czestochowa, Poland; ^4^School of Natural Resource Management, College of Post Graduate Studies in Agricultural Sciences, Central Agricultural University (Imphal), Imphal, India; ^5^Department of Plant Pathology, Faculty of Agriculture, Minia University, Minya, Egypt; ^6^Director of Research, Central Agricultural University (Imphal), Imphal, India; ^7^Agricultural College and Research Institute, Killikulam, TNAU, Vallanadu, India; ^8^Department of Plant Pathology, College of Agricultural and Environmental Sciences, University of California, Davis, Davis, CA, United States; ^9^Department of Plant Pathology, Biswanath College of Agriculture, Assam Agricultural University, Biswanath Chariali, India

**Keywords:** nanotechnology, nanophytovirology, diagnostics, plant disease, management

## Abstract

Plant viruses cause enormous losses in agricultural production accounting for about 47% of the total overall crop losses caused by plant pathogens. More than 50% of the emerging plant diseases are reported to be caused by viruses, which are inevitable or unmanageable. Therefore, it is essential to devise novel and effective management strategies to combat the losses caused by the plant virus in economically important crops. Nanotechnology presents a new tendency against the increasing challenges in the diagnosis and management of plant viruses as well as plant health. The application of nanotechnology in plant virology, known as nanophytovirology, includes disease diagnostics, drug delivery, genetic transformation, therapeutants, plant defense induction, and bio-stimulation; however, it is still in the nascent stage. The unique physicochemical properties of particles in the nanoscale allow greater interaction and it may knock out the virus particles. Thus, it opens up a novel arena for the management of plant viral diseases. The main objective of this review is to focus on the mounting collection of tools and techniques involved in the viral disease diagnosis and management and to elucidate their mode of action along with toxicological concerns.

## Introduction

Agriculture is a basic science that deals with the scientific production of crops to meet world hunger. The world population is expanding rapidly and is expected to reach 10 billion by 2050 (Gill and Garg, 2014); therefore, increasing crop productivity is the utmost concern of all the countries. Improvement in quality and quantity are the prime objectives associated with food production. However, different biotic and abiotic factors cause a considerable loss in the global food production. Pathogens like bacteria, fungi, and viruses are very common biotic factors that are encountered during crop production. Since the time humans understood the destructions that could be done by a pathogen, they have adopted several tactics to avoid them. Many countries enforced an embargo on economically important agricultural crops and products such as grains, which effectively prevented the introduction and spread of disease and many insect vectors (Almeida, 2018). Chemical, biological, and integrated pest management practices are adopted with a vision of minimizing the food loss and maximizing the profit. Despite all these measures, crops are still subjected to infection by many bacterial, fungal, and viral pathogens as well as infestation by various insect pests. Globally, 20–40% of the total agricultural produce is damaged due to pest attacks (Gullino et al., 2021), of which 16% of the yield loss is incurred by plant pathogens (Ficke et al., 2018). In India, potential crop loss due to infection by diseases, pests, and weeds has been estimated at around 15–25% of the annual food production (Kumar et al., 2021). Plant virus being the smallest entities in size serves as a major constraint in crop production. Out of the global food production loss due to plant diseases, viral diseases account for 47% of this loss (Anderson et al., 2004; Popp and Hantos, 2007; Boualem et al., 2016). Phyto viruses cause an array of symptoms due to changes at physiological, molecular, and biochemical levels of the host plant, which is manifested as stunting, chlorosis, distortion, and mottling of fruits and leaves of diseased plants that make them unmarketable and incur loss through a reduction in the growth and yield of the crop. Furthermore, infection by viral pathogens can drastically affect the flowering and fruit settings. Sunflower necrosis caused by the *Tobacco streak virus* (TSV) is the most widespread and destructive viral disease affecting sunflower crops in India and other sunflower growing countries (Prasada Rao et al., 2000; Kumar et al., 2006; Jain et al., 2008). Geminiviruses cause considerable losses in economically important crops like cotton, legumes, tomato, okra, chili, etc. (Reddy and Zehr, 2004; Biswas et al., 2005, 2015). However, the field management of viral diseases is very complex, as most of the economically important plant viruses are transmitted through insect vectors. Therefore, to manage a viral disease successfully, it is essential to stop the transmission of viruses by the insect vectors (Khurana and Marwal, 2016; Marwal and Gaur, 2019). The use of chemical pesticides in bulk has caused tremendous harm to the environment. In this context, the use of nanotechnology might bring forward a positive change. Nanotechnology is one of the fastest developing sciences over the last few years and the potential of nanoparticles (NPs) are emerging rapidly. Nanoparticles have marvelous applications in the field of high sensitivity biomolecular detection, disease diagnostics, and antimicrobial and therapeutic compounds, and therefore likely to improve agricultural productivity due to the low cost associated with agricultural production practices (Dutta, 2012; Dutta et al., 2021). The advantage of NPs lies in their extremely small size (<100 nm), maximum surface area, and strong reactivity, which favors their wide-scale application in plant and human pathology (Jeevanandam et al., 2018). The use of nano-encapsulated pesticides can reduce the quantity of pesticide consumption as compared to conventional pesticides; at the same time, might provide longer protection to various crops. NPs as carrier molecules can release more active ingredients owing to their greater active surface area. Target specificity is also enhanced by nanomaterials, thereby reducing the ill effects on the non-target organisms. Furthermore, NPs can improve plant health and can also increase the disease tolerance ability of plants. Therefore, it may be predicted that in the near future it would be possible to establish integrity in the disease diagnosis and disease management by exploiting the science of nanotechnology (Mahmood et al., 2017). The science of nanophytovirology is still in its nascent stage. Recent studies have shown promising results that indicate NP as a potential antiviral agent along with plant defense activators. In this review, we are going to focus on all the aspects of nanotechnology in viral disease management, thereby summarizing the scattered literature in one place.

## Nanotechnology

Nanotechnology refers to the science of creation, manipulation, and implementation of materials and devices at the molecular, atomic, or supramolecular level (Drexler, 1986). The generalized description of nanotechnology was given by the National Nanotechnology Initiative as “the understanding and control of matter at dimensions between approximately 1 and 100 nm, where unique phenomena enable novel applications”. Nanoparticles exhibit unique physicochemical properties such as improved thermal conductivity, chemical stability, catalytic reactivity, and biological activity as compared to their bulk counterparts owing to their large surface area to volume ratio (Taniguchi, 1974; Edelstein and Cammaratra, 1996). Nanoparticles have marvelous applications in the field of high sensitivity biomolecular detection, disease diagnostics, and antimicrobial and therapeutic compounds (Prasad, 2009). The integration of biological science with nanotechnology is the recent and most potential science of the modern era. Bio-nanotechnology is the conjunction between biotechnology and nanotechnology, which aims at developing environment-friendly and biosynthetic approaches for the synthesis of nano-based materials (Sobha and Surendranath, 2010).

Nanomaterials can be used in the field of plant protection as a protectant or as a carrier for precise and targeted delivery *via* adsorption, encapsulation, or conjugation with active ingredients of pesticides. Nanomaterials can be applied as a foliar spray in seed treatment and root application, as they are known to possess antifungal, antibacterial, and antiviral properties (Wong and Liu, 2010; Krishnaraj et al., 2012; Elbeshehy et al., 2015; Pasha et al., 2022). The NPs exhibiting plant disease suppressing activity include nonmetals, metalloids, metallic oxides, and carbon nanomaterials (Elmer et al., 2018). Silver, copper, zinc oxide, gold, titanium oxide, iron, and more specifically silver are the most popularly studied and used NPs (Wang and Xia, 2004; Iravani et al., 2014; Kandi and Kandi, 2015). The NPs have been found effective against numerous phytopathogenic microorganisms *viz., Rhizoctonia solani, Fusarium oxysporum, Sclerotinia sclerotiorum, Sclerotium rolfsii* (Kaman and Dutta, 2019; Abdelrhim et al., 2021)*, Sarocladium oryzae* (Das and Dutta, 2021), *Colletotrichum* sp. (Goswami et al., 2020), *Xanthomonas oryzae* pv. *oryzae* (Abdallah et al., 2019; Ogunyemi et al., 2020), and viruses such *as Bean Yellow Mosaic Virus* (BYMV) (Elbeshehy et al., 2015), *Cucumber Mosaic Virus* (CMV) (El-Sawy et al., 2017)*, Potato Virus Y* (PVY) (El-Dougdoug and El-Dougdoug, 2018), etc. The mechanism of action of nanomaterials against organisms includes disruption of the cell membrane, agglutination, inhibition of H^+^-ATPase activity, inhibition of synthesis of messenger RNA, proteins, inhibition of toxin production, microbial growth, and blockage of the nutrient flow (Dakal et al., 2016; Malerba and Cerana, 2016). Nanofertilizers are also another important aspect of plant health materials and bear the potential to boost plant disease resistance. It has been explored on numerous plant/disease systems *viz*., Fusarium wilt in chrysanthemum, Fusarium crown, root rot in asparagus, Verticillium wilt in brinjal, red root rot in tea, Fusarium wilt in tomato etc. (Elmer et al., 2018).

## Nanophytovirology

Nanophytovirology refers to the use of nanotechnology for the detection, diagnosis, and management of viruses and their vectors especially arthropods at an initial level to protect plants against economically important viral diseases (Marwal and Gaur, 2019). The plant diseases caused by viruses are difficult to manage as rapid spread of the disease is caused by vectors, mainly arthropods. The increasing spread of viruses causes a threat to the agricultural sector. Therefore, there is a great demand for improved management of viruses employed by a series of strategies that mainly relied on the virus ecology. Many approaches have been used to decrease crop losses caused by viruses; however, only a few are effective in their management. To tackle the viral diseases, understanding the interactions among plants, viruses, and their vector is very crucial. Nanotechnological-based diagnostics and management practices are gaining momentum nowadays to curb the threat caused by plant viral diseases due to their ease in handling and cost-effectiveness (Marwal and Gaur, 2019). Nano-based materials offer advantages like reduced toxicity, enhanced shelf life, and increased solubility of poorly soluble pesticides, all of which provide positive environmental effects (Worrall et al., 2018). There are two aspects for the use of NPs in plant disease management: (i) NPs alone acting as protectants; (ii) NPs as nanocarriers for pesticides and RNA-interference molecules. Nanoparticles can also be used as biostimulants or they can be used precisely in the detection of plant viruses using nano-biosensors.

## Nano-biosensors for the Detection of Plant Viruses

The identification of viruses is very crucial in disease diagnosis and for the formulation of effective management practices. Conventionally, staining of viruses was done using the fluorescent dyes, but nowadays, quantum dots (QDs) and NPs have been developed that help in carrying the detection tags (dyes or anti-viral antibodies), which are efficient tools for the identification of viruses and helpful in imaging and labeling (Cheng et al., 2009). Such fluorescent tags can be detected using flow cytometry-enabled devices. The first attempt of using NPs for virus detection was made by Zehbe et al. (1997), where AuNPs were coupled with silver staining for the detection of human papillomavirus in cervical carcinoma cells. NPs can be conjugated with probes (nucleic acids, antibodies, or aptamers) for the detection of virus-derived molecules upon binding (Rosi and Mirkin, 2005; Dougan et al., 2007; Wang et al., 2009). Nano-biosensors can also be used for the detection of vectors that act as carriers of viruses. For instance, a nano-biosensor was developed to detect the plasmodiophoromycete vector of beet necrotic yellow vein virus (BNYVV), i.e., *Polymyxa betae*, the causal agent of rhizomania disease in sugarcane crop. The biosensor was developed using specific antibodies conjugated with Cadmium-Telluride QDs and glutathione-S-transferase protein's (GST). The developed nano-biosensor exhibits fluorescence resonance energy transfer (FRET) for the detection of the plasmodiophoromycetes vector *Polymyxa betae* (Safarpour et al., 2012). Cadmium-Telluride QDs conjugated with antibodies were developed using a similar approach to detect the most dreaded viral pathogen of the citrus industry, i.e., *Citrus tristeza virus* (CTV) (Marwal and Sahu, 2014). Lavanya and Arun (2021) developed a visual detection method based on functionalized gold NPs containing clccpi1 probe for the detection of begomoviruses in tomato, chili plants, common gram, green gram, and black gram plants. The AuNP assay (77.7%) exhibited better detection results than the conventional PCR technique (49.4%).

Surface plasmon resonance (SPR) is another technique for the detection and identification of viruses based on the changes in the refractive index of a metal surface. In this technique, gold NPs were conjugated with antiviral antibodies adsorbed on a glass substrate and were detected using SPR (Taghipour et al., 2018). Another well-known example of a commercially accessible mass sensor technique is quartz-crystal microbalance (QCM), which is generally used for quantitative measurement of the thickness of thin films. The mechanism includes a quartz-crystal device (dimensions in nanometer) coated with anti-viral antibodies against the targeted plant viruses. When the device comes in contact with a virus, it increases the mass, thereby decreasing the resonant frequency. The changes in the resonant frequency are measured subsequently and thereby the virus is detected (Eun et al., 2002). QCM- and SPR-based nano-biosensor has been manufactured for the detection of various plant viruses such as *Tomato mosaic virus* (TMV), *Cymbidium mosaic virus* (CymMV), *Odontoglossum ringspot virus* (ORSV), and many orchid viruses (Shojaei et al., 2016).

Microcantilevers are another important tool used for the biosensing of various pathogens. Their size lies in the range of micrometer, however, their tip has a nanometer dimension. The two modes involved in the working of microcantilevers are resonating and straining modes used for the identification of viruses. The resonating mode works on the principle similar to QCM, whereas the straining mode detects changes in electrical resistance when the virus particle gets adsorbed on the surface. A major drawback of the device is its low performance in the liquid medium, thus the sample needs thorough drying before application (Chalklen et al., 2020). Nanowires employed transistors have been developed to detect virus particles. Several immuno-biosensors have been devised to detect phytoviruses *viz*., Plum pox virus in the leaves of host plants such as plum (*Prunus domestica*) and tobacco (*Nicotiana benthamiana*). The device was developed using modified gold electrodes using AuNPs, 1,6-hexanedithiol, anti-PPV IgG polyclonal antibody, and bovine serum albumin (Jarocka et al., 2011). When nanowires come in contact with the charged viral capsid of the target virus, a change in charge is recorded as a simple change in conductance. A similar technique *viz*., lithographically patterned nanowire electrodeposition (LPNE) was developed as label-free chemi-resistive sensors based on polypyrrole (PPy) nanoribbon conjugated with anti-viral antibodies to target an important phytovirus *viz*., CMV (Hayden et al., 2003).

The majority of studies on the use of NPs as nanobiosensors depict the utilization of QDs and AuNPs. QDs possess photoluminescence property, which presents them as a suitable candidate for imaging and labeling. Also, QDs are size-tunable and resistant to photobleaching as generally observed in organic dyes that are used for staining, and QDs can also be easily integrated with antibodies, nucleic acids, and oligonucleotides as conferred from the above discussion. Again, AuNPs are positively charged that allows greater interaction with the negatively charged viral particles, and their high electron conduction ability allows the detection of low titer of virus particles. Furthermore, studies must be channelized toward the determination of alternate NPs that can be used for the detection of virus particles in ultra-low concentrations. Also, the focus should be given to the development of universal NP–antibody conjugate that can detect both DNA- and RNA viruses.

Furthermore, the NP-based particle bombardment system mediated gene guns can be used for virus detection and infectivity assay (Abubakar et al., 2019). This technique also allows the establishment of transient or stable gene expression systems to understand the interaction between plant and virus at the cellular level (Acanda et al., 2019). In addition, research must be channelized toward the development and modification of biosensors for greater selectivity, affinity, and efficacy.

## Nanoparticles for the Management of Plant Virus

### Nanoparticles as Inhibitors of Phytoviruses

Nanoparticles are known to exhibit viricidal activity against a broad spectrum of viruses and reduce viral infectivity (Galdiero et al., 2011). In medicine, NPs emerged as a noble therapeutic agent effective in suppressing viral pathogens such as monkey poxvirus *Monkeypox virus* (MPV) (Rogers et al., 2008), herpes simplex virus (HSV) (Baram-Pinto et al., 2010), HIV (Lara et al., 2010), hepatitis B virus (HBV) (Lu et al., 2008), and new castle viral disease (NDV) in cattle (Avilala and Golla, 2019). In the field of agriculture, the potential of NPs has been assessed against numerous phytopathogenic viral pathogens and found encouraging results (Table 1). In India, Jain (2014) proved AgNPs to be an effective antiviral agent and showed complete suppression of *Sunhemp rosette virus* (SHRV) disease on cluster bean after spraying an aqueous solution of AgNPs @50 ppm. Elbeshehy et al. (2015) biosynthesized AgNPs using newly isolated strains of *B. pumilus, B. persicus*, and *B. licheniformis* and confirmed *in vitro* antiviral activity against BYMV infection. Again, they observed that AgNP treatment after 24-h post-infection prevented all the destructive symptoms caused by BYMV. Also, a decline in the concentration of virus, percent infection, and disease severity were observed in the treated plants. However, when NPs were applied at the pre-viral infection stage, no effect was observed. The result suggested that AgNP activity is more profound in the early phases of viral replication as it activates the systemic resistance of the treated plant against BYMV. The inhibitory effects of AgNPs @ 0.1μg/μL alone, and a mixture of AgNPs @ 0.1μg/μL and salicylic acid (SA) @ 200 μmol/L on PVY infected potato plants were observed, and the application of NPs before 3–7 days of virus infection showed a decrease in the virus concentration and percent infection with a significant increase in the plant growth and tuber yield (El-Shazly et al., 2017). When AgNPs were sprayed onto bean leaves, complete suppression of the sun-hemp rosette virus (SHRV) was observed (Jain, 2014). The antiviral effect of AgNPs against TMV and *Potato virus Y* (PVY) in tomato was reported by El-Dougdoug and El-Dougdoug (2018). AgNPs @ 50 ppm suppressed the viral disease severity and percent infection inducing systemic acquired resistance (SAR) against TMV and PVY in tomatoes by increasing the levels of total soluble proteins (TSP), antioxidant enzymes peroxidase (POD), and polyphenol oxidase (PPO) activities. A significant decrease in photosynthetic pigments and an increase in total soluble phenols and free proline were detected in TMV- and PVY-infected tomato plant, respectively (El-Dougdoug and El-Dougdoug, 2018). They observed a reduction in the disease severity caused by *Tomato yellow leaf curl virus* (TYLCV) by delaying the viral symptom production in treated tomato plant as compared with non-treated control plants. The activities of POD and PPO increased in SiNPs-treated tomato plants, indicating enhanced systemic resistance against TYLCV (El-Sawy et al., 2018). ZnONPs were also observed to enhance resistance against CMV in brinjal plants under greenhouse conditions (El-Sawy et al., 2017). Similarly, gold NPs were found effective against plant pathogenic viruses *viz*., bean mild mosaic virus infecting beans, tobacco necrosis virus in tobacco, and barley yellow mosaic virus infecting barley plants. Ghosh et al. (2018) first reported *in planta* efficacy of two antimicrobial agents *viz*., 2S albumin-a plant-based protein, Nano-Zinc oxide (Nano-ZnO), and their combinations against the fastidious bacterial pathogen, *Candidatus Liberibacter asiaticus* (*Ca*.Las) causing Haunglongbing (HLB) disease in citrus. The application of 2S albumin and Nano-ZnO formulation alone or in various combinations showed a marked reduction in *Ca*.Las titers in affected plants as compared to untreated HLB-infected citrus plants. Thus, it can be concluded from the above discussion that NPs alone can be utilized as an effective antimicrobial agent to curb the menace caused by plant viruses.

**Table 1:** Antiviral properties of potential nanoparticles.

**Nanoparticle**	**Size**	**Target virus**	**Host plant**	**Effect**	**Reference**
Silver nanoparticles (AgNPs)	12.6 ± 5 nm	Tomato spotted wilt virus (TSWV)	*Solanum tuberosum*	Inhibition of local lesions and reduction in TSWV infection	Shafie et al. (2018)
AgNPs	12 nm	Potato virus Y (PVY)	*S. tuberosum*	Induction of resistance in host plant against virus	El-Shazly et al. (2017)
Schiff base nanosilver NPs	-	TMV	*Nicotiana tabacum*	Reduction in infection caused by TMV	Wang et al. (2016)
Zinc oxide NPs (ZnONPs)	18 nm	Tobacco mosaic virus (TMV)	*Nicotiana benthamiana*	Suppression of speed of TMV invasion	Cai et al. (2019)
Iron oxide NPs (Fe_3_O4)	0.19 nm	TMV	*N. benthamiana*	Induction of plant resistance and activation of antioxidants against TMV by upregulation of SA genes	Cai et al. (2019)
Graphene oxide-silver NPs (GO-AgNPs)	3050 nm	Tomato bushy stunt virus (TBSV)	*Lactuca sativa*	Decrease in virus concentration, infection percentage, and disease severity	Elazzazy et al. (2017)
Gold NPs (AuNPs)	-	Barley yellow mosaic virus (BaYMV)	*H. vulgare*	Dissociation of viral particles *in vitro*	Aref et al. (2012)
Silicon dioxide NPs (SiO_2_NPs)	100 nm	Tomato yellow leaf curl virus (TYLCV)	*Solanum lycopersicum*	Reduction in disease severity and TYLCV concentration	El-Shazly et al. (2017)
Carbon Nanotubes (CNTs)	-	TMV	*N. benthamiana*	TMV movement and replication inhibition and induction of resistance.	Adeel et al. (2021)
Titanium dioxide NPs (TiO_2_NPs)	~3–5 μm	Broad bean stain virus (BBSV)	*Vicia faba* L. Fabaceae	Reduction in disease severity	Elsharkaway and Derbalah (2018)
Nickel oxide NPs (NiONPs)	15–20 nm	Cucumber mosaic virus (CMV)	*Cucumis sativus*	Reduction in disease severity and CMV concentration	Derbalah and Elsharkawy (2019)
Cerium oxide NPs (CeO_2_NPs)	–	TMV	*Datura stramonium* and *Nicotiana tabacum*	Suppression of viral symptoms	Eugene and Zholobak (2016)

Chitosan NPs were also found to induce resistance against a few viruses in host plants *viz*., the mosaic virus of potato, peanut, cucumber, alfalfa, and snuff. Murphy et al. (2003) evaluated the effect of combinations of two PGPR strains formulated with chitosan molecule known as preparations, and observed a significant reduction in disease severity with significantly higher plant height, root and shoot weight, and a number of flowers and fruits in treated plants as compared to control. Thus, it was confirmed that tomato plants treated with biopreparations exhibited enhanced plant growth with greater protection against CMV infection. The antiviral activity of graphene-based silver nanocomposites was confirmed against *Tomato bushy stunt virus* (TBSV) by reducing the concentration of virus and disease severity in lettuce plant in case of both soil and foliar application (Elazzazy et al., 2017). SiO_2_NPs treated *via* soil supplementation caused a significant decline in symptom production by *Papaya ringspot virus* (PRSV) infected *Cucumis sativus*. Also, a decline in *Broad bean strain virus* (BBSV) infection was observed when TiO_2_NPs were applied using spray and soil drench after 4 h of inoculation.

From the above discussion, we can infer that metal and metal-oxide NPs have been widely studied for their antiviral properties, as they possess broad-spectrum antimicrobial effects. It is established from the previous studies that NPs interfere mainly during the early virus replication cycle and induce SAR in plants. However, further studies must be conducted to determine the exact mode of action of NPs on viral movement, replication, encapsidation, and transmission. Also, the effects of nanocomposites and a combination of organic polymer-based nanomaterials and metallic NPs against complex viral diseases need to be studied for the effective management of viral diseases under field conditions.

The inhibitory mechanisms followed by nanoparticles may include the release of metal ions followed by oxidative and other non-oxidative stresses. The underlying mechanism involved includes the inactivation and denaturation of capsid protein, nucleic acids (RNA or DNA), and other protein molecules. The NPs may also prevent virion binding, fusion, infectivity, and replication. Reactive oxygen species (ROS) generation and subsequent oxidation of proteins lead to inactivity and virucidal activity. The NPs may also interfere with the virus recognition and subsequent entry into the host plant. The NPs interact with virus surface proteins *via* glycoprotein receptors, and thus interferes with virus recognition by the host cells. Cai et al. (2019) studied the *in vitro* effect of ZnONPs and SiO_2_NPs against the phytovirus TMV. The result suggested that direct inactivation of TMV by MeNPs is caused by the interaction of NPs with glycoprotein envelope, which leads to direct injury of TMV shell protein, affects the aggregation, and may even cause fracture. Fe_3_O_4_NPs-treated TMV particles also exhibit similar aggregation and a fracture pattern (Cai et al., 2020). AgNPs can also adhere to capsid protein of PVY and ToMV (El-Dougdoug and El-Dougdoug, 2018). In the case of barley yellow dwarf virus-PAV, puffed virus-like particles decorated with gold NPs and destroyed and vanished virus particles were observed using TEM (Alkubaisi and Aref, 2017).

Several *in vivo* studies have also been conducted to assess the effectiveness of MeNPs against viral infections in different plant families of crops, Poaceae, Cucurbitaceae, Fabaceae, Asteraceae, and Solanaceae (Vargas-Hernandez et al., 2020). The applied NPs interfere with the replication process of viruses through numerous mechanisms. Again, in the case of CeO_2_NPs, the replication of the virus was affected after penetration into the TMV-infected leaves of *N. tabacum* and *Datura stramonium via* a vascular system of plants (Eugene and Zholobak, 2016).

### Nanoparticles as a Carrier of DsRNA for Inducing Gene Silencing

The two engrossing sciences of the twenty-first century are nanotechnology and RNAi; these technologies when coupled together bear the potential to revolutionize the field of plant disease management. RNAi refers to the technique that provides resistance against endogenous parasitic or exogenous pathogenic nucleic acids *via* dsRNA molecules by altering mRNA stability and protein translation (Hannon, 2002). Here, NPs can be loaded with dsRNA or other viral protein molecules such as coat protein to induce resistance in plants against the target virus. NPs allow target-specific delivery of RNAi agents with greater probability of internalization by the target plant cells/tissues. A notable study was carried out to exhibit resistance against *Pepper mild mottle virus* (PMMoV) and CMV. In this technique, layered double hydroxides (LDH) were loaded with dsRNA called as BioClay and were sprayed on the challenged plants. The plants were found to exhibit resistance against the above-stated viruses for 20 days as compared to the control (Elbeshehy et al., 2015). Worrall et al. (2018) used topical application of dsRNA to induce RNA interference, and it was found effective in a range of plants. They found that topical application of dsRNA was effective against aphid-mediated inoculation and mechanical inoculation with *Bean common mosaic virus* (BCMV). The topical application of dsRNA targeting either the coatprotein coding region (BCMVCP-dsRNA) or the potyviral nuclear inclusion b (Nib) coding region that protected the tobacco (*N. benthamiana)* and cowpea (*Vigna unguiculata*) plants against mechanical inoculation with BCMV. BCMVCP was loaded onto LDH NPs to form BCMVCP–BioClay. Further study demonstrated topical application of dsRNA using BioClay protects both the host plants from aphid-mediated BCMV transmission, which paves the way toward practical application of this approach for crop protection.

Nanomaterials can also be used as the carrier for the delivery of antiviral agents such as nanoliposomes that are being used as carrier molecules for biological antiviral molecule quercetin. Hsp70 is the target gene for quercetin through biotic and abiotic stress, and nanoliposomes enhanced the inhibitory effect by 33.6% and 42.0%, at the gene and protein levels, respectively. Field trials also showed higher control efficacy (increased by 38%) than conventional quercetin formulations. The study suggests that the use of nanomaterials will enhance the efficacy as well as reduce the dose of active ingredients required for the effective management of the disease (Wang et al., 2022).

## Viral Inhibition *VIA* Induction of Plant Defense Mechanisms in Plants

### Antioxidant System

The plant defense response to various stresses includes the generation of ROS that limits the spread of the pathogen and induces local and systemic defense responses such as the release of pathogenesis-related (PR) proteins. The ROS generation in plants is induced by several biotic and abiotic stimuli. When the level of ROS rises above the threshold, oxidative products are generated and the equilibrium between ROS and antioxidants is disrupted. The effect of oxidants is countered by the antioxidant system in plants. The antioxidant system consists of enzymes such as superoxide dismutase (SOD), ascorbate peroxidase (APX), catalase (CAT), and guaiacol peroxidase (GPX) (Tan et al., 2018). NPs affect the cellular redox homeostasis by either exciting or reducing the occurrence of oxidative stress (Soares et al., 2018). Previous studies suggest that NPs possess the ability to either induce the production of ROS or repress the oxidative burst in the host plant depending upon the plant requirement. The entry of a virus into the plant system causes a rapid burst of ROS that affects the host cells, in such case, NPs induce an antioxidant system to repress the oxidative burst. For instance, CeO_2_, CuO, ZnO, TiO_2_, Fe_3_O_4_, Al_2_O_3_, γ-Fe_2_O_3_ NPs, CoFe_2_O_4_, Ag, and NiO can induce an antioxidant system and repress the oxidative burst in various crops (Soares et al., 2018). ZnO, Fe_3_O_4_, and SiO_2_ NPs also enhanced the activity of antioxidant enzymes such as CAT and POD. Several other enzymes were also expressed in presence of the virus. For instance, cucumber plants treated with SiO_2_NP led to the expression of pox and pal gene expression after 24 h of PSRV inoculation (Elsharkawy and Mousa, 2015). Again, cucumber plants treated with NiONPs post 4 days of CMV inoculation increased expression of the pod gene (Derbalah and Elsharkawy, 2019). On the other hand, NPs can sensitize the plants to produce ROS as a defense mechanism prior to the entry of the virus particles. Cai et al. (2019) confirmed the induction of ROS in Fe_3_O_4_NPs-treated tobacco leaves applied by foliar spray. The induction of ROS could be related to the induction of resistance in tobacco leaves. The application of ZnO and SiO_2_ NPs induces the accumulation of hydrogen peroxide even in the absence of the virus.

### Plant Hormones and Pathogenesis-Related Proteins

The resistance in plants operates mainly through an extensive defense mechanism network, wherein the signaling molecules *viz*., SA, jasmonic acid (JA), and ethylene (ET) trigger appropriate defense responses. Other plant growth regulating hormones bearing the potential to regulate defense responses include gibberellin (GA), cytokinin (CK), auxin [indole-3-acetic acid (IAA)], abscisic acid (ABA), brassinosteroids (BRs), and strigolactone (SL). The equilibrium between plant growth and defenses is regulated by crosstalk between multiple plant hormones. NPs are known to influence the plant hormonal balance (Rastogi et al., 2017). Landa et al. (2017) reported elevated concentrations of SA and JA along with the transcription of genes involved in defense signaling in *Arabidopsis thaliana* upon exposure to CuONPs. Again, the mixture of polyvinyl alcohol hydrogels (Cs-PVA) and CuNPs leads to overexpression of the JA gene in tomato plants under salt stress (Hernández-Hernández et al., 2018). Cai et al. (2020) observed elevated SA and ABA levels in *N. benthamiana*, while no change was recorded in the JA level. Thus, the above results suggest that the expression of a particular plant hormone depends on peculiar interaction between NPs and plants, time, and dose of application. The expression of other hormones such as CK was also found to increase in *Capsicum annuum* L. upon exposure to AgNPs (Vinković et al., 2017). Again, ZnONPs in a small dose caused an increase in CK biosynthesis, and at moderate and high doses, they may also act as a stressor molecule that upregulates plant defenses and the level of stress hormones *viz*., SA and ABA (Landa et al., 2017). Plants use several mechanisms to modulate plant growth and regulate defense mechanisms against plant pathogens including viruses. Tobacco plant infected with TMV upon exposure to TiO_2_ NPs and Fe_2_O_3_ alters the level of phytohormones such as ABA, zeratin riboside, and brassinoid (Hao et al., 2018). Furthermore, tomato plants exhibited an increase in proline content upon being treated with AgNPs (El-Dougdoug and El-Dougdoug, 2018). Another important component of a plant's immune system includes PR proteins, which act as part of diagnostic molecular markers of plant defense signaling pathways. The activation of PR1, PR2, and PR5 genes indicate upregulation of the SA signaling pathway (Ali et al., 2018). The uninfected tobacco plants (*N. benthamiana*) exposed to SiO_2_ NPs and ZnONPs upregulate SA-inducible PR genes, PR1 and PR2, and a similar effect was observed due to the treatment of Fe_3_O_4_NPs (Cai et al., 2020). Thus, altered levels of phytohormones and PR proteins in NP-treated plants indicate activation of the antiviral plant defense mechanism.

### Secondary Metabolite Production

Several studies have been conducted to determine the effect of NPs on secondary metabolite production in plants, however, the mechanism is still not clearly understood. According to some authors, ROS production due to interaction with NPs interferes with the plant's secondary metabolite production (Marslin and Sheeba, 2017). The enzyme, phenylalanine ammonia-lyase (PAL) is considered a bridge point between primary and secondary metabolism. It is produced during biotic and abiotic stress conditions and induces SA/JA production. The enzyme PAL catalyzes non-oxidative deamination of phenylalanine to trans-cinnamate. SiO_2_NP treatment in PRSV-infected plants, as well as NiONP-treated CMV infected plants, showed an increase in JA-induced PAL (Elsharkawy and Mousa, 2015; Derbalah and Elsharkawy, 2019). Chung et al. (2019) studied the effect of CuONPs and observed upregulation of the *Pal* gene with enhanced phytochemicals *viz*., phenolics and glucosinolates in *Brassica rapa*. A similar result was shown in tobacco plants with PAL enzyme activity along with flavonoid and phenolic compound accumulation upon exposure to ZnONPs (Mazaheri-Tirani et al., 2019). ZnONPs also enhanced the anthocyanin and total phenolic content in potato plants (Raigond et al., 2017).

### Nanoparticles as Biostimulants in Virus-Infected Plants

Plant biostimulants can be defined as substances and materials other than pesticides and plant nutrients, bearing the potential to modify physiological and biochemical processes in plants in such a way that would stimulate photosynthesis, plant growth, development, and defense mechanisms (du Jardin, 2015). The studies conducted on healthy tobacco plants treated with ZnO, SiO${}_{2}^{,}$ and Fe_2_O_3_ NPs found an increase in plant growth (Cai et al., 2019, 2020). Thus, NPs can cause eustress effects in healthy as well as stressed plants (for instance, virus-infected plants), as they have the potential to counteract the harmful effects caused by them. Derbalah and Elsharkawy (2019) reported that foliar application in infected cucumber plants and soil drenching with NiONPs exhibited greater fresh and dry weight as well as an increase in the number of leaves (Derbalah and Elsharkawy, 2019). Again, TuMV-infected tobacco plants when treated with 50 mg/L of FeO_3_ NPs and TiO_2_ showed an increase in fresh and dry weight, whereas treatment with 200 mg/L had no significant difference as compared with control plants without nanoparticle treatment (Hao et al., 2018). El-Shazly et al. (2017) reported improved-quality potato tubers in AgNP-treated PVY-infected plants as compared to infected plants without treatment. The possible reason might be the induction of resistance or hindrance in virus entry due to the application of NPs. It can also be used to prevent virus contamination in seeds. Seed transmission of viruses occurs through contamination of seed coat or by an invasion of the embryo of seeds before fertilization or directly from the mother plant to embryonic tissues after fertilization or indirectly from infection of plant gametes (Cobos et al., 2019). The NPs can be used as seed antivirals by limiting the pathogen in the seed due to their size and antimicrobial properties. NPs can also be used to prevent seedling contamination, whereas *in vitro* culture is the most successful approach for eradication of the virus in seedlings (Wang et al., 2016). Further studies revealed that the antiviral activity of NPs is more prominent in cultures before viral infection (Cai et al., 2019, 2020). Thus, NPs are proposed as preventive control measures that can be used for seed or seedling treatment at an early stage to protect against viruses, fungi, or bacteria (Sánchez-López et al., 2020).

## Challenges Associated With Nanophytovirology

In a national forum, nanotechnology has been described as multidisciplinary science of manipulating matters at the nanoscale with novel physical, chemical, and biological properties. It can be easily understood that designing a nanoparticle requires very specific structural and physicochemical properties. Any variation in size and structure can affect the function and performance of the designed nanoparticle conjugate (Mitter et al., 2017). Therefore, several factors are kept under strict supervision during the synthesis of NPs for the development of effective assays of virus detection. During the process of synthesis, it remains a challenging task to maintain their uniformity in terms of shape and size. Further, the NPs alone cannot detect the virus particles in the plant body, and therefore there is a need for additional biomolecules for sensing the pathogen. However, these biomolecules are quite sensitive to severe chemical and physical alterations such as high salt concentrations, reducing agents, high temperature, etc., which might affect their reactivity and specificity (Spicer et al., 2018). Therefore, proper optimization steps for such procedures are required for the successful detection of viruses.

At the time of application of nanopesticides for viral disease management, their biosafety and toxicity to human health and the environment is a major concern. Sometimes, the nanocomposites might get deposited on the leaves or flowers, which can affect animals, birds, honey bees, etc. They may clog the stomatal pores and might hinder the penetration of pollen grains on stigma. Cellular toxicity induced by NPs can lead to toxic side effects such as enhanced ROS generation, disruption of redox homeostasis, lipid peroxidation, impaired mitochondrial function, and membrane damage (Marwal and Gaur, 2019). The interaction of nanoformulations with other pollutants present in the environment is an important area that requires research. For a particular nanopesticide, the degree of toxicity is directly related to the surface and surface properties of these nanoformulations that can create adverse effects on microorganisms, plants, and humans at different trophic levels. Several studies are carried out to understand the fate, behavior, and effects of nanoformulations in the soil in terms of their mobility and transformation under different environmental conditions (Lewis et al., 2016; Queyrel et al., 2016). The toxicity of a nanoformulation in any plant system is influenced by its uptake, translocation, and biotransformation pathways involved in the plant system. In an experiment with PEG-coated imidacloprid nanoformulation, Adak et al. (2012) found less accumulation of nanopesticide on the soybean seeds and soil than conventional pesticides. For the successful adoption and/or commercialization of the nanoformulations, it is crucial to properly account for the concerns and risk perception from the consumer perspective, while considering different aspects of effective risk communication at the same time.

## Conclusion

Plant pathogenic viruses represent a highly destructive group of plant pathogens that cause significant crop loss due to their genetic diversity, rapid multiplication, evolution, and lack of effective management options. Nanotechnology has emerged as a potential management approach to curb the menace caused by plant viral diseases. The unique physicochemical properties of NPs allow better interaction with viruses, vectors, and their host plants in numerous beneficial ways. NPs can directly interact with virus particles and cause physical inhibition and destruction. The indirect interaction may cause activation of antiviral plant defense mechanism and biostimulation of host plants. NPs can be conjugated with various probes for the development of nano-biosensors that can be used for the detection and diagnosis of phytoviruses. Nano-based materials can also be developed as carrier molecules for target-specific delivery of dsRNA for gene silencing in phytoviruses. However, more study is needed to gain further insight into the mechanism of interaction between plant-virus vectors and NPs. Nanophytovirology is still in its nascent stage and requires extensive research in this field. Furthermore, composites of NPs with other nutrients, pest management approaches, or biostimulants should be investigated as management strategies. Also, the probability of implementation of NP-mediated management strategy of plant virus and vectors as a component of integrated disease management should be explored. The wholesome development of nanophytovirology requires the combined effort of researchers from multiple disciplines such as plant pathologists, biologists, and agricultural engineers. Thus, efforts must be focused on the development of a sustainable, effective, safer, long-lasting, and eco-friendly management approach against plant viral diseases.

## Future Prospects

Nanophytovirology presents a promising tool for the sustainable protection of crops against plant viruses. Although, several studies have indicated the potential of NPs for the management of plant viruses based on *in vitro* studies. Further *in vivo* trials are essential to obtain comprehensive results for future use. Again, a majority of the studies have been conducted on RNA viruses while DNA viruses continue to pose a persistent threat toward crop production. Moreover, the disease management strategies are devised based on the interaction of components of the disease cycle. Plant viral disease occurs as a result of interaction between plant-virus–vector–host-environment. Thus, quadripartite interaction makes the process very complex and difficult to analyze. The interaction of NPs with plant-virus–vector–host still remains unknown. The queries related to the complex interaction include the exact mechanism of interaction of NPs with virus particles, and the effect of NPs on the acquisition, transmission, and movement of vectors. For instance, AuNPs affect the reproduction and development of insects by inhibiting trypsin (Patil et al., 2016), while other metal and metal oxide NPs specifically bind with phosphorous and sulfur in proteins and nucleic acids leading to impaired membrane permeability (Jiang et al., 2015; Benelli et al., 2018). Again, NPs in many cases found to be effective against viruses if they are applied as prophylactically in host plants, while in some others, it was found effective when applied postinoculation of viruses. Thus, further work is needed to get a deeper insight into the host–virus interaction. Again, a majority of the studies are based on single NPs; however, nanocomposites can be proved path-breaking in the management of complex viral diseases. Nanocomposites may allow targeting multiple plant viruses simultaneously, thus providing greater benefits. Also, the effects of NPs on satellite viruses or other infectious RNA molecules are yet to be explored. The potential of nano-based materials as a component of integrated viral disease management needs to be evaluated. However, the major concern remains the use of NPs in the field as regulatory and legislative measures still need to be developed.

## Author Contributions

PD, AK, and MMah: conception or design of the work and drafting the work. KB, AA, AD, GM, KS, MMan, NM, and DT: revising it critically for important intellectual content. All authors read and finalized the article.

## Conflict of Interest

The authors declare that the research was conducted in the absence of any commercial or financial relationships that could be construed as a potential conflict of interest.

## Publisher's Note

All claims expressed in this article are solely those of the authors and do not necessarily represent those of their affiliated organizations, or those of the publisher, the editors and the reviewers. Any product that may be evaluated in this article, or claim that may be made by its manufacturer, is not guaranteed or endorsed by the publisher.
